# L’infarctus du myocarde du jeune adulte -Analyse rétrospective des cas colligés au CHU de Dakar

**Published:** 2010-09-29

**Authors:** Yameogo Nobila Valentin, Ndiaye Mouhamadou Bamba, Mbaye Alassane, Bennani Rajaa, Kagambega Larissa Justine, Bodian Malick, Diao Maboury, Sarr Moustapha, Kane Abdoul, Ba Serigne Abdou

**Affiliations:** 1 CHU Aristide le Dantec, Dakar, Sénégal; 2 Hôpital Général de Grand Yoff, Dakar, Sénégal

**Keywords:** Infarctus du myocarde, facteurs de risque cardio-vasculaire, jeune adulte, Afrique noire, Sénégal

## Abstract

**Abstract:**

Les données relatives à l’infarctus du myocarde chez le jeune adulte sont rares en Afrique noir. Nous rapportons une série rétrospective de 14 cas dninfarctus du myocarde chez l’adulte jeune noir africain. Pour analyser les caractéristiques épidémiologiques, cliniques, électriques, échographiques, biologiques, thérapeutiques et évolutives de l’infarctus aigu du myocarde chez le jeune adulte nous avons étudié de manière rétrospective les dossiers médicaux d’une série consécutive des patients admis entre Janvier 2003 et Décembre 2008 pour prise en charge d’infarctus aigu du myocarde. Quatre-vingt quatre (84) cas d’infarctus du myocarde ont été pris en charge durant la période d’étude ,14 patients (16,6%) avaient un âge inférieur ou égal à 40 ans. L’âge moyen était de 34 +ou-5ans (extrêmes 27ans et 40 ans). Les facteurs de risque cardiovasculaire étaient dominés par le sexe masculin (n=11), la dyslipidémie (n=7) et le tabagisme par cigarette (n=6). La contraception orale était absente chez le 1/3 des patients. Le délai moyen d’admission était tardif (15 plus ou moins 4 heures). L’IDM était antérieur dans la moitié des cas. L’acide acétylsalicylique, les inhibiteurs de l’enzyme de conversion Les bétabloquants et les statines étaient les médicaments les plus prescrits. La thrombolyse et l’angioplastie étaient respectivement réalisées chez 3 patients et 0 patient. 4 cas d’insuffisance cardiaque, 2 cas de bloc atrioventriculaire complet et 1 cas de décès étaient les principales complications. L’infarctus du myocarde concerne également le sujet jeune noir africain. Les facteurs de risque sont essentiellement représentés par le sexe, la dyslipidémie, le tabagisme et la contraception orale.

## Introduction

L’infarctus du myocarde (IDM) du sujet jeune constitue une entité singulière et si les accidents coronaires aigus sont inhabituels sur ce terrain, ils ne sont pas exceptionnels [1,2]. Les mécanismes responsables sont incomplètement élucidés, mais à côté de l'athérosclérose qui est en la cause la plus fréquente, d'autres mécanismes plus rares peuvent être à l'origine [2-10]. Les données relatives à l’infarctus du myocarde chez le jeune adulte sont rares en Afrique noire [8]. L'infarctus du myocarde est essentiellement décrit chez les sujets adultes [6,7]. Nous rapportons dans ce travail une série rétrospective de 14 cas d'infarctus aigu du myocarde chez le sujet noir africain d’âge inférieur ou égal à 40 ans, dans un service de cardiologie au Sénégal.

L’objectif de notre étude était d’analyser les caractéristiques épidémiologiques, cliniques, électriques, échographiques, biologiques, thérapeutiques et évolutives de l’infarctus aigu du myocarde chez le jeune adulte pris en charge au Centre Hospitalier et Universitaire (CHU) de Dakar.

## Méthodes

Cette étude documentaire a analysé de manière rétrospective les dossiers médicaux d’une série consécutive des patients admis entre Janvier 2003 et Décembre 2008 pour prise en charge d’infarctus aigu du myocarde. Le service de cardiologie du CHU de Dakar, Sénégal a servi de cadre d’étude.

Les paramètres d’intérêt étaient les suivants: être noir, la proportion des jeunes adultes (inferieur à 40 ans) et leurs caractéristiques épidémiologiques (facteurs de risque cardiovasculaire dont le sexe, l’hérédité, la dyslipidémie et le tabagisme), cliniques (douleur thoracique typique, les circonstances de découverte, le diagnostic Clinique d’infarctus), électriques (sus-décalage du segment ST, localisation d’ondes Q), biologiques (élévation de CK-MB et de la troponine), échocardiographiques ( akinésie myocardique et réduction de la fraction d’éjection), thérapeutiques (médicaments et thrombolyse) et évolutives (complications et décès).

## Résultats

Parmi l’ensemble de 84 cas d’infarctus du myocarde pris en charge durant la période d’étude ,14 patients (16,6%) avaient un âge inférieur ou égal à 40 ans. L’âge moyen était de 34 +ou-5ans (extrêmes 27ans et 40 ans).

Parmi les facteurs de risque cardio-vasculaire, le sexe masculin (n=11), la dyslipidémie (n=7) et le tabagisme par cigarette (n=6) étaient plus fréquents que les autres facteurs de risque cardiovasculaire (3 cas d’hypertension artérielle, 1 cas de diabète sucré, 1 cas d’obésité et 1 cas de sédentarité). La contraception orale était absente chez le 1/3 des patients. Le délai moyen d’admission était tardif (15 plus ou moins 4 heures). La moitié des patients (n=12) présentaient les signes électriques d’infarctus aigu du myocarde à localisation antérieure. L’acide acétylsalicylique, les inhibiteurs de l’enzyme de conversion, les bétabloquants et les statines étaient les médicaments les plus prescrits. La thrombolyse et l’angioplastie étaient respectivement réalisées chez 3 patients et 0 patient. Les proportions des médicaments prescrits en hospitalisation et à la sortie d’hôpital sont représentées dans le Tableau 1. Les complications étaient rares: 4 cas d’insuffisance cardiaque, 2 cas de bloc atrio-ventriculaire complet et 1 cas de décès. Le séjour moyen en Unité de Soins Intensifs Cardiologiques était de 6 plus ou moins 3 jours.

## Discussion

La petite taille de notre échantillon et l'absence de données coronarographiques constituent les deux limites de ce travail. Néanmoins il garde son intérêt car à notre connaissance il fait partie des toutes premières séries publiées chez l’adulte jeune noir africain.

Les syndromes coronaires aigus constituent un enjeu majeur de santé publique [12,13]. Cependant, l’infarctus du myocarde du sujet jeune est plus rare mais son incidence est sans cesse croissante [2,9,14]. En Afrique où la réalité des maladies cardio-vasculaires est plus que jamais prouvée [6], nous disposons de peu de données épidémiologiques sur l'infarctus du myocarde de l’adulte jeune. Ce travail constitue, à notre connaissance, l’une des premières séries africaines publiées sur la question. Nous avons retrouvé une prévalence plus élevée que celle rapportée dans la plus part des études antérieures où elle a été évaluée à 4 à 10 % [1,2,10,11,15,16]. Une étude japonaise a trouvé une prévalence de 1,6 % [17]. Cette disparité des résultats pourrait être liée aux critères utilisés dans la définition de l’adulte jeune. En effet certaines études ont considéré la limite d’âge de 40 ans sans distinction de sexe [11,17] mais d’autres ont utilisé l'âge de 45 ans. [2,9,10,15,18]. D’autres encore ont considéré la limite d'âge de 45 ans pour les hommes et 55 ans pour les femmes [9].

La prédominance masculine est constante dans l'infarctus du sujet jeune ce qui est le cas dans notre série. D’autres études ont montré une progression croissante de la maladie chez la femme jeune [9,16].

La proportion des femmes dans notre étude, 21,4% (soit 3 patientes) semble s'inscrire dans cette tendance. Cette augmentation de la maladie dans la frange jeune de la population serait très probablement en rapport avec l’augmentation des facteurs de risque modifiables dans ladite population [9]. En effet, hormis le sexe masculin et l'hérédité familiale de maladie coronaire, tous les autres facteurs de risque retrouvés sont modifiables. Ce fait lié à l'origine athéromateuse prédominante de la maladie [19] prend toute son importance dans la prévention car des moyens efficaces de prise en charge de ces facteurs de risque sont disponibles avec un bénéfice prouvé en termes de mortalité cardio-vasculaire [9,20]. Parmi ces facteurs de risque, toutes les études s'accordent sur le fait que tabagisme en est le principal [1,2,9-12,15,17,19]. Ce facteur vient en troisième position dans notre série. Cette faible prévalence du tabagisme dans notre série pourrait être expliquée en partie par la différence des habitudes de vie et des croyances socioculturelles. Les autres facteurs de risque (obésité, hypertension artérielle, sédentarité) sont en général retrouvés dans des proportions inférieures. Toutefois, dans la pathogénie de l'IDM de l’adulte jeune, d'autres mécanismes sont décrits impliquant les anomalies congénitales des artères coronaires, les états d'hypercoagulabilité et l'utilisation de certaines drogues [3-5,13,19].

Le pronostic à court terme de l'infarctus du myocarde du sujet jeune est réputé meilleur que celui du sujet plus âgé notamment en cas de prise en charge précoce par angioplastie coronaire en particulier chez les patients ayant une atteinte mono-tronculaire [2,11,13-15,19]. Le suivi à plus long terme confirme ce bon pronostic mais l'effet favorable initial semble s'estomper au delà de 9 ans avec une augmentation de la mortalité [21,22]. La maladie peut entraîner dans une certaine mesure une morbidité importante [13,14] notamment dans les conditions de travail difficiles.

La mortalité était de 7,14 % (1/14) dans notre étude. L'amélioration du pronostic à long terme nécessite en plus du traitement conventionnel, une bonne éducation du patient en particulier pour l'arrêt du tabac chez les fumeurs. A ce sujet, Jacquemin et al [14] rapportent une adhésion correcte au traitement préventif secondaire pour les principales classes thérapeutiques mais avec un fort taux d'échec du sevrage tabagique sur le long terme.

## Conclusion

La place importante et la réalité de l’infarctus aigu du myocarde de l’adulte jeune noir Africain ont été démontrées dans cette petite série des patients. Un tiers de cas ne présentaient pas de douleur thoracique typique d’infarctus aigu du myocarde. La prévention doit vite se focaliser autour du sexe masculin, les pilules contraceptives chez la femme, la dyslipidémie et le tabagisme par cigarettes.

## Conflits d’intérêts

Les auteurs ne déclarent aucun conflit d’intérêts.

## Figures and Tables

**Tableau 1:** Pourcentage des médicaments administrés aux 14 jeunes adultes hospitalisés au CHU de Dakar pour infarctus du myocarde de 2003 à 2008

**Médicaments**	**Pourcentage (%)**
Aspirine	92,8
Clopidogrel	42,8
Bêta bloquant	78,6
Inhibiteur de l’enzyme de conversion	85,7
Statine	64,3
Dérivés nitrés	28,5
Trimétazidine	14,2
Inhibiteur calcique	7,4
Streptokinase	37,7
Anti-vitamine K	21,4
